# Development and Validation of a New Reliable Method for the Diagnosis of Avian Botulism

**DOI:** 10.1371/journal.pone.0169640

**Published:** 2017-01-11

**Authors:** Caroline Le Maréchal, Sandra Rouxel, Valentine Ballan, Emmanuelle Houard, Typhaine Poezevara, Marie-Hélène Bayon-Auboyer, Rozenn Souillard, Hervé Morvan, Marie-Agnès Baudouard, Cédric Woudstra, Christelle Mazuet, Sophie Le Bouquin, Patrick Fach, Michel Popoff, Marianne Chemaly

**Affiliations:** 1 ANSES, Laboratoire de Ploufragan – Plouzané, Unité Hygiène et qualité des produits avicoles et porcins, Université Bretagne-Loire, Ploufragan, France; 2 Labocea, Ploufragan, France; 3 ANSES, Laboratoire de Ploufragan – Plouzané, Unité d’Epidémiologie et bien-être en aviculture et cuniculture, Université Bretagne-Loire, Ploufragan, France; 4 ANSES, Laboratoire de sécurité des aliments, Maisons Alfort, France; 5 Institut Pasteur, Bactéries anaérobies et Toxines, Paris, France; The University of Melbourne, AUSTRALIA

## Abstract

Liver is a reliable matrix for laboratory confirmation of avian botulism using real-time PCR. Here, we developed, optimized, and validated the analytical steps preceding PCR to maximize the detection of *Clostridium botulinum* group III in avian liver. These pre-PCR steps included enrichment incubation of the whole liver (maximum 25 g) at 37°C for at least 24 h in an anaerobic chamber and DNA extraction using an enzymatic digestion step followed by a DNA purification step. Conditions of sample storage before analysis appear to have a strong effect on the detection of group III *C*. *botulinum* strains and our results recommend storage at temperatures below -18°C. Short-term storage at 5°C is possible for up to 24 h, but a decrease in sensitivity was observed at 48 h of storage at this temperature. Analysis of whole livers (maximum 25 g) is required and pooling samples before enrichment culturing must be avoided. Pooling is however possible before or after DNA extraction under certain conditions. Whole livers should be 10-fold diluted in enrichment medium and homogenized using a Pulsifier^®^ blender (Microgen, Surrey, UK) instead of a conventional paddle blender. Spiked liver samples showed a limit of detection of 5 spores/g liver for types C and D and 250 spores/g for type E. Using the method developed here, the analysis of 268 samples from 73 suspected outbreaks showed 100% specificity and 95.35% sensitivity compared with other PCR-based methods considered as reference. The mosaic type C/D was the most common neurotoxin type found in examined samples, which included both wild and domestic birds.

## Introduction

Avian botulism has become an emerging issue in Europe in recent years [1]. It induces high mortality and morbidity in waterfowl and poultry during outbreaks and causes significant economic losses. Clinical symptoms include progressive paralysis, with limberneck as typical sign [2]. Botulism is caused by botulinum neurotoxins (BoNT), which act at the neuromuscular junction and inhibit neurotransmitter (i.e. acetylcholine) release in cholinergic nerves by interfering with the exocytosis mechanism, leading to flaccid paralysis. The clinical symptoms are indicative but not specific for botulism and laboratory confirmation is required to validate the diagnosis.

The predominant BoNT involved in recent European avian botulism outbreaks is the mosaic type C/D [2–4]. Outbreaks due to types D, D/C or C have been also reported but at a lower rate [3, 4]. *Clostridium botulinum*, which produces BoNT is divided into four physiological groups. Strains producing types C, D, C/D or D/C BoNTs belong to physiological group III. Physiological group II strains that produce BoNT type E can also be involved in avian botulism outbreaks. Outbreaks are reported annually in the Great Lakes area in North America [5–10] and some outbreaks were reported in France in the 1990’s on poultry farms [11]. Although no type E outbreak has been diagnosed in France since 2001, this BoNT type is still systematically included in avian botulism testing due to the zoonosis risk [11]. Detection and typing of types C, D, C/D, D/C and E are helpful for epidemiological investigations and crucial for implementing appropriate outbreak management measures.

We recently showed that liver is the best matrix to use for detecting *C*. *botulinum* types C, D, C/D, D/C and E using real-time PCR [3]. However, the steps preceding PCR have not yet been optimized. The aim of this study was 1) to optimize the pre-PCR steps to maximize the sensitivity and specificity of the diagnosis method and 2) to validate the diagnosis method by determining its limit of detection (LOD) and its sensitivity and specificity according to the recommendations of the NF U 47–600 standard [12, 13]. Sample test and minimal incubation period were optimized using naturally contaminated samples; and storage conditions, homogenization method, incubation temperature, and anaerobic conditions were optimized using spiking samples. Both naturally contaminated and spiked samples were used for the comparison of DNA extraction methods. Finally, the diagnosis method developed here was also applied to identify the BoNT type involved in recent avian botulism outbreaks.

## Materials and Methods

### Optimization of the method using naturally contaminated samples

In 2015, veterinarians were asked to send livers from five birds with clinical symptoms per suspicion for laboratory testing of suspected avian botulism outbreaks. As far as possible, samples were collected from shortly euthanized birds and not from bird carcasses. Clinical botulism suspicions were based on typical indicative signs such as flaccid paralysis and high mortality. The number of samples sent per suspicion varied among veterinarians, and depended on availability of sample material. Both wild birds and domestic poultry birds were included in this study.

**Fig 1:**
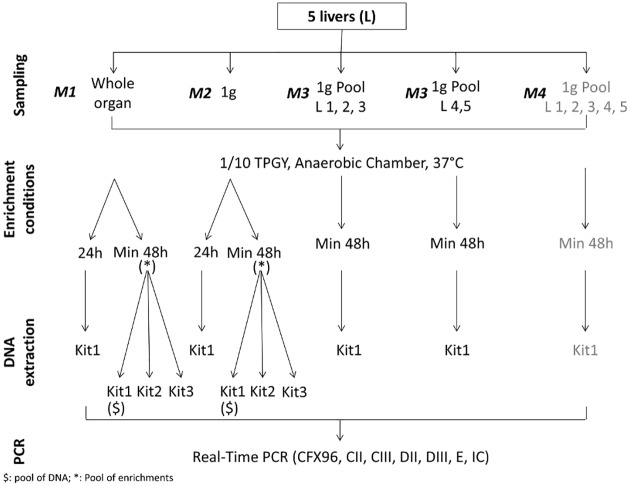
Sample analysis workflow for suspected avian botulism cultures. Parameters evaluated during the study: test sample type (whole organ means that the whole liver or up to 25g of the liver was analyzed), enrichment culturing conditions, DNA extraction methods. The CFX96 thermocycler (Bio-Rad, Marne-la-Coquette, France) was used for Real-Time PCR. Analyses performed by ANSES are indicated in black, and performed by LABOCEA are in grey. Kit 1: QIAamp^®^ DNA Mini kit (Qiagen, Courtaboeuf, France), Kit2: InstaGene Matrix (Bio-Rad, Marne-la-Coquette, France), Kit3: Mericon Bacteria+ (Qiagen, Courtaboeuf, France). CII, CIII, DII, DIII, E are the primers and probe used to perform real-time PCR for the detection of type C, D, C/D, D/C and E BoNT genes [4, 14]. L: Liver; L1 to L5: Liver N° 1 to Liver N° 5. M1 to M4: Method 1 to method 4.

Samples were analyzed using the five test sample strategies depicted in Fig 1 and detailed below.

LABOCEA (testing laboratory accredited for performing analyses for botulism diagnosis confirmation) performed the test on 1 g of pooled livers from the same suspicion (method 1) (see below and Fig 1) and the French National Reference Laboratory for avian botulism (ANSES Laboratoire de Ploufragan-Plouzané) performed the three other tests (Methods 2 to 4).

Several types of samples were tested:

- Method 1: Analysis of 1 g of pooled liver samples from the same suspicion (M1 in Fig 1)
  A slice from each liver was sampled and pooled to constitute a sample of 1 g. This pool of five livers was 10-fold diluted in pre-reduced tryptone, peptone, glucose yeast extract (TPGY) broth [15] and incubated for at least 48 h (incubation lasted 48h at minimum or longer) at 37°C in an anaerobic chamber (Anaerobic workstation concept 300, Ruskinn, distributed by Oxoid & Remel Microbiology, Dardilly, France). This analysis was performed by LABOCEA.
- Method 2: Analysis of 1 g of two or three pooled livers (M2 in Fig 1)
  Livers were manually processed individually in BagFilter^®^ blender bags (Biomerieux, Craponne, France). Aliquots of two (or three) processed livers were pooled to obtain a 1 g sample and then diluted in 9 ml of pre-reduced TPGY. After homogenization using a vortex, the pooled sample was incubated for at least 48 h at 37°C in an anaerobic chamber (A35, Don Whitley distributed by Biomérieux, Bruz, France).
- Method 3: Analysis of 1 g of each individual liver(M3 in Fig 1)
  An aliquot of 1 g of each processed liver (Biomerieux, Craponne, France) was diluted in 9 ml of pre-reduced TPGY. After homogenization using a vortex, they were incubated at least 48 h at 37°C in an anaerobic chamber (A35, Don Withley distributed by Biomérieux, Bruz, France).
- Method 4: Analysis of individual livers (M4 in Fig 1)
  The remaining same livers were finally weighed in BagFilter^®^ blender bags (Biomerieux, Craponne, France), and a maximum of 25 g was 10-fold diluted in pre-reduced TPGY and homogenized using a Pulsifier^®^ blender (Microgen, Surrey, UK) for 15 s. The sample was then incubated for at least 48 h at 37°C in an anaerobic chamber (A35, Don Withley distributed by Biomérieux, Bruz, France) for enrichment. When feasible, a 1 ml aliquot of each enrichment culture was sampled after 24 h of incubation.

Gas used in the anaerobic chambers consisted of 10% CO_2_, 10% H_2_ and 80% N_2_ (Air Liquide, Jay, France).

After incubation, 1 ml of the enriched sample was collected. Cells were pelleted by centrifugation and used for DNA extraction using QIAamp^®^ DNA Mini kit (Qiagen, Courtaboeuf, France) according to the manufacturer's instructions. Two other DNA extraction methods were tested according to the manufacturer's instructions: InstaGene Matrix (Bio-Rad, Marne-la-Coquette, France), Mericon Bacteria+ (Qiagen, Courtaboeuf, France). These methods were selected because they are quick and/or cheap.

Finally, the possibility of pooling samples after the enrichment step (1 ml and 200 μL of each individual enrichment was collected and pooled per outbreak) or after DNA extraction (20 μl of each DNA extract was collected and pooled per outbreak) was evaluated.

### Optimization of the method using spiked samples

Livers from broilers Ross PM3 were crushed using a blender and distributed in BagFilter^®^ blender bags (5g of livers per bag) (Biomerieux, Craponne, France). Chicken livers (5 g) were spiked with a known concentration of *C*. *botulinum* spores to study some parameters of the method (storage conditions, sample homogenization, enrichment incubation conditions and DNA extraction kits) and evaluate the method’s LOD. Spore solutions from *C*. *botulinum* type C (strain CIP-109 785, from the collection housed at the Institute Pasteur, France), type D (strain CIP-105 256, Institute Pasteur, France) and type E (strain HV, CNR anaerobic bacteria and botulism, Institute Pasteur, France) were prepared and titrated by the five-tube most-probable number (MPN), as previously described (Lindberg et al. 2010, De Man 1983). Samples of chicken liver (5 g) that tested negative for the presence of *C*. *botulinum* using real-time PCR were inoculated with different numbers of spores of each strain.

Spiked samples were 10-fold diluted in pre-reduced TPGY broth, processed using a Pulsifier^®^ blender (Microgen, Surrey, UK) for 15 s and incubated for at least 24 h under anaerobic conditions. A volume of 1 ml was then used to carry out DNA extraction using the QiaAmp^®^ DNA Minikit (Qiagen, Courtaboeuf, France).

All experiments were performed in at least six replicates.

Different times and temperatures of sample storage before analysis were tested using spiked samples: 7 days at 20°C; 24 h, 48 h and 7 days at 5°C; 7 days, 1 month and 6 months at a temperature below -18°C.

Two blenders for homogenization before incubation were tested: the Pulsifier^®^ blender (Microgen, Surrey, UK) for 15 s and a paddle blender at 240 rpm (Mix1, AES, Bruz, France) for 1 min.

Several incubation temperatures were also tested: 30°C, 37°C and 41.5°C.

Finally, methods to produce anaerobic conditions were tested: anaerobic chamber (A35, Don Whitley distributed by Biomérieux, Bruz, France), anaerobe container system using either Gas-pak (AnaeroGen, Oxoid, Dardilly, France) or the same gas used for the anaerobic chamber (10% CO_2_, 10% H_2_ and 80% N_2_).

Two other DNA extraction methods were tested: DNeasy Blood and Tissue kit (Qiagen, Courtaboeuf, France) and Nucleospin Tissue (Macherey-Nagel, Hoerdt, France).

### Real-time polymerase chain reaction

Real-time PCR, primers and probes used in this study were selected according to Woudstra et al. [4]. Real-time PCR using a Bio-Rad CFX96 thermal cycler (Bio-Rad,) was used to detect *C*. *botulinum* types C, C/D, D, D/C and E. Each assay was performed in a total volume of 20 μl, containing 5 μl DNA template, 10 μl IQ supermix (Bio-Rad, Marne-la-Coquette, France) and a final concentration of 600 nM for primers and 400 nM for probes. The thermal profile consisted of 5 min at 95°C, followed by 40 cycles of denaturation at 95°C for 15 s and annealing/elongation at 55°C for 30 s. Primers and probes used were: CII, CII, DII, DIII [4] and E [14]. A PCR positive for CII and CIII means a type C, positive for CII and DIII means a type C/D, positive for CIII and DII means a type D/C, positive for DII and DIII means a type D [4] and positive foe E means a type E.

Each PCR run included positive and negative controls for each target and a commercial internal amplification control (QuantiFast Pathogen + IC Kits; Qiagen, Courtaboeuf, France) was used according to the manufacturer's instructions. A sample was considered positive when the Ct (Cycle threshold) was below 38.

### Data analysis

Regarding naturally contaminated samples, a suspicion was considered confirmed when *C*. *botulinum* was detected in at least one sample, regardless of the method used for detection. In particular regarding the method 3, a suspicion was considered positive when at least one of the pools was positive. This series of tests was primarily used to assess the possibility of pooling samples at different pre-PCR stages and to compare results obtained on pooled and unpooled samples.

Regarding spiked samples, 10-fold dilutions of type C or D spores were used. Only results obtained for samples spiked with 50 spores/g of liver and 5 spores/g of liver are presented here. At least six replicates were performed for each condition.

Diagnostic sensitivity and specificity of the method developed in this study were evaluated according to the NF U 47-600-2 standard [12, 13] by comparing the results obtained with the method developed here and other methods considered as reference methods [16, 17]:

Sensitivity=True Positive/(True positive + False negative)

Specificity=True negative/(True negative + False positive)

Two different levels were considered for the evaluation of diagnostic sensitivity and specificity: the individual sample level and the suspected outbreak level.

## Results

### Optimal test sample for detection of *C*. *botulinum* in avian livers

Thirty-four suspected outbreaks were included in the test sample comparison, of which 22 were confirmed avian botulism outbreaks: 18 type C/D, 1 types C and C/D, 1 types C/D and D, 1 type D and 1 type D/C.

**Fig 2:**
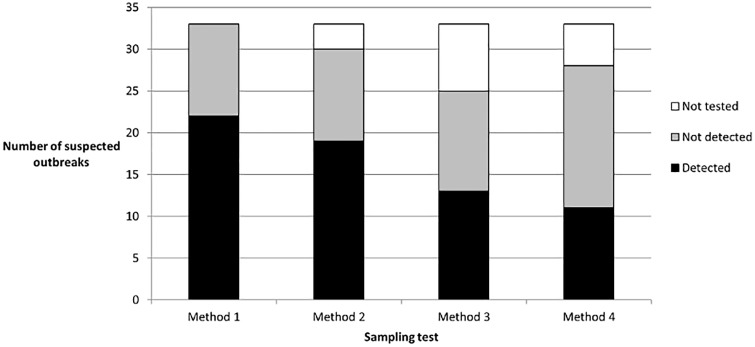
Confirmation of avian botulism suspicions using different types of test samples. Method 1: the entire liver was analyzed (max 25 g), Method 2: 1 g of each single liver was analyzed, Method 3: an aliquot of each liver was sampled after its homogenization in Bagfilter^®^ (Biomérieux, Craponne, France) and separated into two groups and 1 g of both pools were analyzed, Method 4: a slice of each liver was sampled and 1 g of this pool was analyzed. For each type of test sample, the number of suspected outbreaks detected is shown in black, the number of suspected outbreaks not detected is shown in gray, and the number of suspected outbreaks not tested is shown in white.

Several types of samples were evaluated to identify the optimal type of test sample for *C*. *botulinum* detection (Fig 1), and to test the possibility of pooling samples. Results obtained by varying test samples before enrichment culturing are shown in Fig 2. Analysis of whole livers (up to 25 g) gave the highest confirmation rate (100%). Analysis of 1 g of liver (not pooled) confirmed only 90% of suspicions: two suspicions were not confirmed by the analysis of this type of test sample. Upon analysis of 1 g of the five pooled livers, 8 outbreaks went undetected among the 19 suspicions confirmed by other test sample methods (58% confirmation rate). Upon analysis of 1 g of two or three pooled livers, 5 outbreaks went undetected among the suspected outbreaks confirmed by other test sample types (72% confirmation rate). These results show that samples must not be pooled at this stage of the diagnosis method.

Whole livers (up to 25 g) should therefore be individually 10-fold diluted and individually cultured for enrichment for optimal detection of *C*. *botulinum* to confirm avian botulism.

### Pooling of samples before or after DNA extraction

Pooling of samples prior to PCR was also evaluated in the interest of reducing the number of DNA extracts for analysis.

Pooling after the enrichment step was tested by pooling 200 μL and 1 ml of each enriched sample, leading to detection of 94% of the confirmed outbreaks. Pooling DNA extracts before PCR by pooling 20 μL of each DNA extract allowed also detection of 94% of the confirmed outbreaks. In either pooling strategy, 6% of the outbreaks were not detected.

Both methods can therefore be applied. However, to avoid false-negative results, each DNA extract or each enriched sample should be individually analyzed for each negative result after pooling.

### Minimal incubation period for *C*. *botulinum* detection in avian livers

Livers underwent enrichment culturing for at least 48 h before DNA extraction. When feasible, an aliquot was collected in the anaerobic chamber after 24 h of incubation. For all samples that were shown to be positive at 48 h, a DNA extraction was also performed on the 24 h aliquot. For 42 samples, the 24 h aliquot was analyzed and a 100% confirmation rate was obtained, showing that an incubation period of 24 h might be sufficient for the detection of *C*. *botulinum* in avian livers.

### Optimal conditions for sample storage before analysis

**Fig 3:**
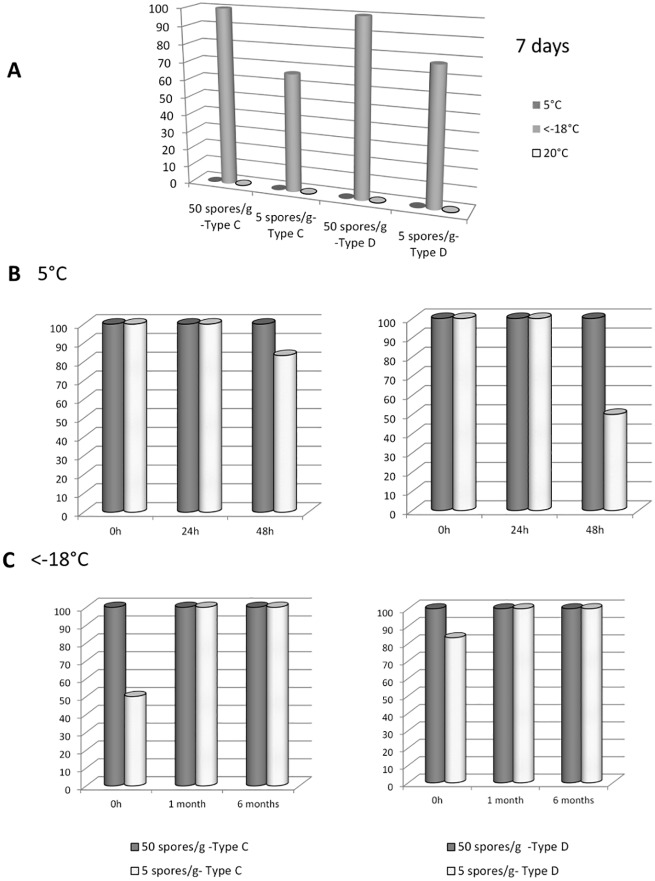
Percentage of detection of 5 or 50 spores of *C*. *botulinum* strain 109 785 (type C) or 105 256 (type D) in spiked liver samples after A) storage of spiked samples for 7 days at 20°C, 5°C or at a temperature below -18°C (n = 9 for each condition); B) storage of spiked samples at 5°C for 0 h, 24 h and 48 h (n = 6 for each condition), C) storage at a temperature below -18°C for 1 month and 6 months (n = 6 for each condition).

Different temperatures (room temperature, 5°C and -18°C) and storage times (24 h, 48, 1 week, 1 month, 6 months) were compared and evaluated with regard to their impact on the detection of *C*. *botulinum* spores. Spiked livers were used for this experiment. Storage at -18°C was the only temperature that allowed detection of *C*. *botulinum* in all spiked samples as shown in Fig 3A, regardless of storage duration (7 days, 5 weeks, 6 months). Storage at 5°C or at 20°C for 7 days hindered the detection of *C*. *botulinum* at the tested dilutions.

Short-term storage at 5°C did not affect the detection of *C*. *botulinum*, as shown in Fig 3B. However, by 48 h, a decrease in the detection level was observed for samples spiked with 5 spores per gram of liver, indicating that although short-term storage (24 h) is possible at 5°C, storage at -18°C is better for long-term storage.

### Comparison of two blenders for homogenization before incubation

**Fig 4:**
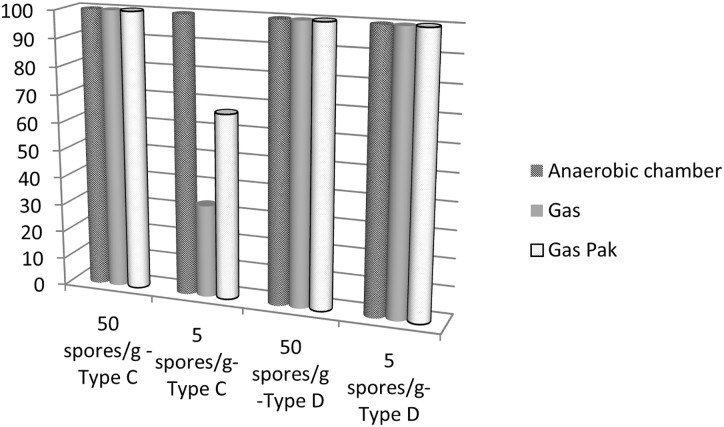
Detection of 5 or 50 spores per gram of liver of *C*. *botulinum* strain 109 785 (type C) or 105 256 (type D) in spiked liver samples after homogenization with either Pulsifier^®^ blender (Microgen, Surrey, UK) (in white) or paddle blender (in gray) (n = 9 for each condition) and after incubation for 24 h in an anaerobic chamber at 37°C. A: detection of *C*. *botulinum* after homogenization with Pulsifier^®^ (Microgen, Surrey, UK) or paddle blender, B: boxplot of the threshold cycle number (Ct) obtained when livers were spiked with 50 spores, C: boxplot of Ct obtained when livers were spiked with 5 spores. CII, CIII, DII, DIII: names of the primers used for the detection of type C (CII, CIII) and type D (DII and DIII) *C*. *botulinum*. P: Pulsifier^®^ blender (Microgen, Surrey, UK), S: Paddle blender.

The use of the Pulsifier^®^ blender (Microgen, Surrey, UK) during 15 s or a paddle blender (Mix1, AES, Bruz, France) for 1 min to homogenize livers in TPGY did not affect the detection rate as shown in Fig 4A: both blenders allowed the detection of 5 spores per gram of liver. However, the type of blender had a significant effect on the obtained Ct values, with a difference of more than 6 Ct for the same sample (Student test, P<0.001), the Pulsifier^®^ blender (Microgen, Surrey, UK) allowing an earlier Ct. Considering that the method developed in this study was qualitative and not quantitative, this difference did not affect our conclusion on presumptive positive determination. Based on this result, the Pulsifier^®^ blender (Microgen, Surrey, UK) should be preferred when both types of homogenizers are available in the laboratory.

### Optimal anaerobic conditions for group III *C*. *botulinum* detection

Three different ways to carry out anaerobic incubation were tested: anaerobic chamber, anaerobic containers filled with gas or Gas-pak. Livers were inoculated at two spore concentrations (50 and 5 spores/g) of type C and D.

**Fig 5:**
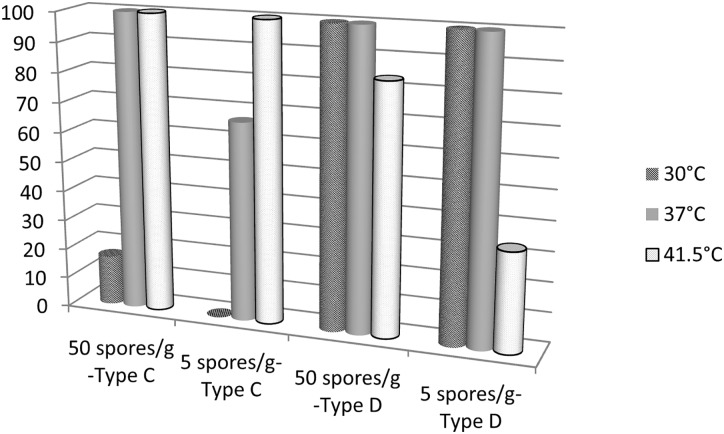
Percentage of detection of 5 or 50 spores of *C*. *botulinum* strain 109 785 (type C) or 105 256 (type D) in spiked liver samples after incubation for 24 h at 37°C in an anaerobic chamber (A35, Don whitley, distributed by Biomérieux, Bruz, France), in an anaerobic container with gas or with a Gas-pak (AnaeroGen, Oxoid, Dardilly, France) (n = 6 for each condition).

The detection rate was the same for samples spiked with 50 spores (Fig 5), regardless of the anaerobic method used. However, a decrease in detection was observed with the anaerobic containers at 5 spores/g for strain CIP 109 785. Anaerobic chambers should therefore be preferred to detect low levels of type C spores. For type D spores, there was no difference between the three different anaerobic systems, indicating that anaerobic containers might be used.

### Optimal temperature for detection of group III *C*. *botulinum*

**Fig 6:**
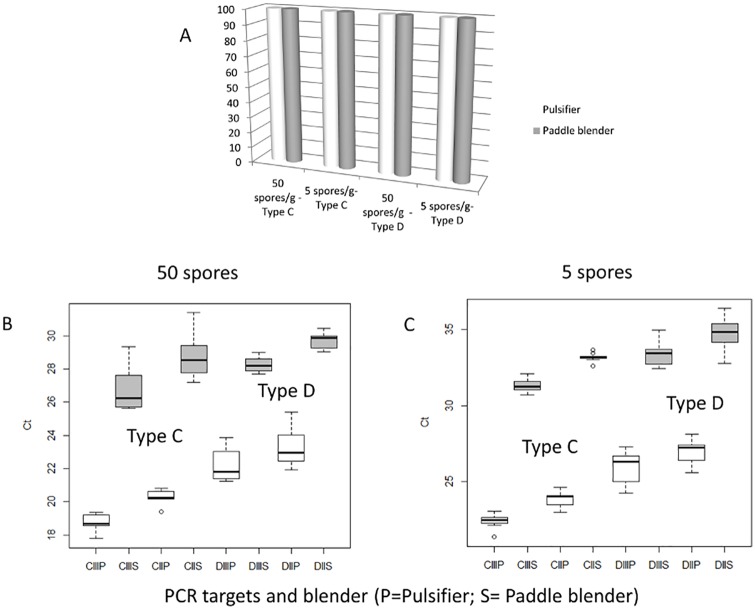
Percentage of detection of 5 or 50 spores of *C*. *botulinum* strain 109 785 (type C) or 105 256 (type D) in spiked liver samples after incubation for 24 h in an anaerobic container with gas at 30°C, 37°C and 41.5°C (n = 6 for each condition).

Different incubation temperatures (30°C, 37°C and 41.5°C) were tested for type C and type D spores. Livers were spiked at two spore concentrations (50 and 5 spores per gram of liver). Results are depicted in Fig 6.

Incubation at 41.5°C gave the best detection rate for type C spores followed by 37°C that gave 100% detection when samples were spiked with 50 spores/g and 67% when spiked with 5 spores/g. However, incubation at 30°C and 37°C gave the best detection rate (100%) for type D spores, whereas incubation at 41.5°C allowed 83% of detection for samples spiked with 50 spores/g and 33% for those spiked with 5 spores.

None of the tested temperatures were optimal for both spiking levels and strains. The consensus temperature for detection of both strains appeared to be 37°C.

### DNA extraction method

Three commercially available DNA extraction methods were tested on 71 naturally contaminated samples after enrichment. A decrease in the *C*. *botulinum* detection rate was observed when using InstaGene Matrix (Bio-Rad, Marne-la-Coquette, France) or Mericon DNA Bacteria Plus kit (Qiagen, Courtaboeuf, France) instead of the QiaAmp^®^ DNA mini kit (Qiagen, Courtaboeuf, France): InstaGene Matrix (Bio-Rad, Marne-la-Coquette, France) allowed only 87.3% of detection and Mericon DNA Bacteria Plus kit (Qiagen, Courtaboeuf, France)) 69% of detection, while QiaAmp^®^ DNA mini kit (Qiagen, Courtaboeuf, France) gave a 100% detection rate on the 71 samples examined. Considering this result, two other commercially available kits based on the same approach (enzymatic digestion and DNA purification on silica columns) were tested on spiked samples: the DNA Blood and Tissue kit and the Nucleospin Tissue (Macherey-Nagel, Hoerdt, France) and both gave a 100% detection. Based on these results, a DNA extraction method including an enzymatic digestion step and a DNA purification on silica column should be used for optimal detection of type C and D spores in avian liver.

### Limit of detection of the method

**Fig 7:**
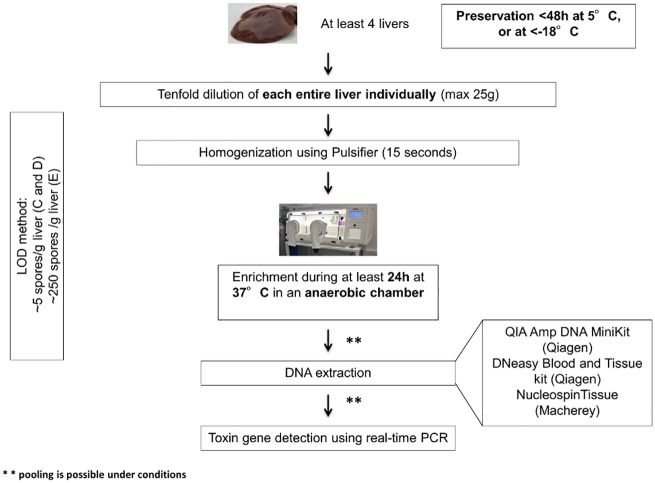
Diagnosis scheme for avian botulism by detection of *C*. *botulinum* in livers using real-time PCR. Parameters optimized in this study are shown in bold.

Chicken livers (5 g) spiked with *C*. *botulinum* spores types C, D and E at different levels, ranging from 1 to 500 spores, were tested using the method depicted in Fig 7. Briefly, the method developed and optimized here consisted in enriching four whole livers 10-fold diluted in TPGY and homogenized using a Pulsifier^®^ blender (Microgen, Surrey, UK) for at least 24 h at 37°C in an anaerobic chamber. After incubation, DNA extraction was performed using a method with enzymatic digestion and DNA purification on silica column and BoNT gene detection using real-time PCR. According to this protocol, the LOD for types C and D was 5 spores/g liver and for type E was 250 spores/g liver (Table 1).

**Table 1:** Detection of toxin type in spiked livers using the method developed in this study (Fig 7).

		Toxin type		
		C	D	E
**Spores (MPN/g liver)**	**500**	**NT**	**NT**	**8+/8**
	**250**	**NT**	**NT**	**8+/8**
	**50**	**8+/8**	**8+/8**	***6+/8***
	**5**	**8+/8**	**8+/8**	***0+/8***
	**<1**	***3+/8***	***5+/8***	**NT**

Livers were inoculated with different levels of spores, type C, D or E. The number of spores was determined by the most-probable number (MPN). NT: not tested, X+/8: number of positive results detected (n = 8).

### Diagnostic specificity and sensitivity of the method

Naturally contaminated livers (n = 268) from avian botulism events reported in France in 2014 and 2015 were analyzed for the presence of *C*. *botulinum* using the method developed in this study (Fig 7). All samples were also tested for the neurotoxin gene profile using two other real-time PCR systems as reference methods [16, 17].

All samples gave positive results for the internal amplification control, showing that the samples carried no significant levels of PCR inhibitors. Out of 268 samples (from 73 suspected outbreaks that occurred in 2014 and 2015), 133 tested positive for the presence of BoNT genes using the method developed here. Of these samples, 93 were type C/D (from 32 outbreaks), 17 were type D/C (from 4 outbreaks), 13 were type D (from 3 outbreaks), 5 (from the same outbreak) were types C and C/D (positive for primers CII, CIII and DIII), 1 was types C/D and D (positive for primers CII, DIII and DII), 4 (from the same outbreak) were positive for all primers. No naturally contaminated type E sample was detected during the study.

Most outbreaks occurred in turkeys (15), mallard ducks (7), guineafowl (6) and broilers (5). The most common BoNT type detected was type C/D, which involved all bird species. Types D and D/C were only found in turkey botulism outbreaks (Table 2).

**Table 2:** Botulinum neurotoxin type detected using the method depicted in Fig 7 in each tested avian species. (Other: duck and sea gull; duck and crow; duck and goose; duck and guineafowl; hen, duck and teal).

		Toxin Type					
		C and C/D	C/D	D	D/C	C/D and D	Positive
**Species**	**Breeding hen**	**\**	**1**	**\**	**\**	**\**	**\**
	**Broiler**	**\**	**4**	**\**	**\**	**1**	**\**
	**Guinea fowl**	**\**	**5**	**\**	**\**	**\**	**1**
	**Mallard duck**	**1**	**6**	**\**	**\**	**\**	**\**
	**Swan**	**\**	**2**	**\**	**\**	**\**	**\**
	**Turkey**	**\**	**8**	**3**	**4**	**\**	**\**
	**Other**	**\**	**6**	**\**	**\**	**\**	**\**

When considering each sample individually (268 liver samples), the sensitivity of the method according to the definition in the NF U 47-600-2 standard was 88.43% [84.7%-92.33%] and specificity was 99.17%.

The analysis of four livers is usually recommended for avian botulism confirmation using real-time PCR [3]. When considering our results on an outbreak basis (i.e. considering the results obtained from the analysis of all samples from the same outbreak and not from each individual sample) (73 suspicions), diagnostic sensitivity was 95.35% [90.52%-100%] and diagnostic specificity was 100%, demonstrating the usefulness of analyzing several samples to confirm a suspicion.

## Discussion

The mouse bioassay is considered as the gold standard to confirm botulism despite many drawbacks: it is cumbersome, low-throughput, time-consuming, expensive, and raises ethical concerns with regard to the use of animals. Laboratory confirmation for animal botulism can be done by different strategies including demonstration of the presence of BoNT or BoNT-producing clostridia in serum, gastrointestinal content, liver and wound [18]. We recently showed statistically that the analysis of four livers to confirm avian botulism using real-time PCR is a reliable method for avian botulism diagnosis [3]. However, the steps preceding the PCR had not been yet optimized to maximize the sensitivity of the method. The aim of this study was thus to optimize the pre-PCR steps to design a reliable and sensitive global method for avian botulism diagnosis.

In numerous other studies, the standard sample size for testing is 1 g [2, 4, 15, 19–22]. However, our results show that the analysis of 1 g of livers was less efficient in confirming botulism than the analysis of whole livers (up to 25 g). The analysis of pooled liver slices also appeared unreliable for the detection of *C*. *botulinum*. These results indicate that contamination of the liver is not homogenous throughout the liver. It is not known whether the botulinum spores always infect the same area within the liver or if the contaminated area of the liver can be identified prior to sampling. However, given that spore growth and BoNT production occur in the cecum [23–25] and that the liver filters venous blood from the spleen and the intestine, spores or vegetative cells may be internalized in Kupffer cells or localized in specific blood vessels in the liver. The pathogenesis of avian botulism, in particular the potential role of *C*. *botulinum* localized in organs in the outcome of the disease is unknown and should be explored.

Pooling of samples is cost- and time-effective. However, for botulism diagnosis, it cannot be implemented before the enrichment step. Pooling after enrichment of each individual liver enriched sample is possible under certain conditions: if a result is negative, every individual sample must be analyzed to rule out the possibility of a false negative.

Storage conditions were evaluated. Surprisingly, they appear to have a strong effect on the detection of *C*. *botulinum*. Storage at 5°C or at room temperature for 1 week reduced the detection of 50 spores of *C*. *botulinum*, whereas storage at a temperature below -18°C during 1 week or more ensures detection. Short-term storage at 5°C is possible, but a decrease in detection was observed as of 48 h. Considering that *C*. *botulinum* spores persist and can be detected in the environment for many years [26–28], storage conditions were expected to have little effect on detectability. This strong storage effect could be attributed to modification of the matrix during storage, such as the release of enzymes that prevent germination and growth of *C*. *botulinum* spores. Therefore, storage at a temperature below -18°C is recommended, and our results might suggest that the longer the storage at this temperature, the better the detection. The storage of samples at a temperature below -18°C is widely used for *C*. *botulinum* detection in animals [19–22, 29, 30]. Furthermore, our results indicate that the interval between sampling and reception at the testing laboratory must be as short as possible.

We tested two blenders that can be used for sample homogenization. There was no difference in detection: both blenders led to the detection at contamination levels of 5 spores per gram of liver. This result is concordant with previous studies, which gave identical bacterial recovery rates [31–34]. However, there was a significant difference in Ct values between the two blenders. The Ct obtained with the paddle blender was significantly later than the Ct value obtained with Pulsifier^®^ blender ((Microgen, Surrey, UK). This can be attributed to a higher quality of microbial suspension obtained when using the Pulsifier^®^ ((Microgen, Surrey, UK)) in comparison with a paddle blender [32] because it is less destructive to the sample than the paddle blender and thus produces less debris. By reducing the disruption of the matrix, it may minimize interference with PCR [33, 34], which could explain the difference in Ct values. Homogenization using the paddle blender was tested for only 1 min here. Longer durations could be tested to evaluate the impact on Ct. However, homogenization using Pulsifier^®^ (Microgen, Surrey, UK) only lasts 15 s which is in any outbreak less time-consuming than the paddle blender. When both are available in a laboratory, the Pulsifier^®^ (Microgen, Surrey, UK) should be preferred.

Conditions of enrichment were evaluated in this study. Incubation of at least 24 h appeared to be sufficient. Similarly, previous reports also propose an incubation time of 24 h [19, 20]. Therefore, diagnosis confirmation time can be shortened, especially in comparison with the mouse bioassay. The use of an anaerobic chamber was also better than the use of an anaerobic container, regardless of the anaerobic atmosphere. Therefore, insofar as possible, the use of an anaerobic chamber for the detection of *C*. *botulinum* group III is recommended, especially for the detection of a low level of spores. The temperature used for enrichment of *C*. *botulinum* group III varies among studies in the literature: 30°C [19, 20, 22], 37°C [2, 15, 35] and 40°C [21, 29]. Here, we compared three different incubation temperatures: 30°C, 37°C, and 41.5°C (Fig 6). Surprisingly, the optimal temperature for the detection of type D strain and type C strain was not the same: 30°C was optimal for type D while 41.5°C was optimal for type C. Detection results obtained at 37°C for the tested type D and C strains were however satisfactory and this temperature represents a good consensus value for detection of group III strains when the toxin type is unknown.

Several DNA extraction methods were tested during this study, on naturally contaminated samples and on spiked samples. Kits were selected according to their price, their ease of use and their speed. Kits including enzymatic lysis steps and purification on silica column gave the best detection results, as demonstrated by Auricchio et al. (2013) on various matrices. In contrast to their result, there was no amplification for some samples when using a Chelex resin-based method (here InstaGene Matrix (Bio-Rad, Marne-La-Coquette, France) made with a 6% w/v Chelex resin was tested) and this extraction method was therefore not selected.

The LOD of the complete method in the matrix liver was evaluated by testing the detection of known concentrations of *C*. *botulinum* spore types C, D and E. The LOD for liver samples spiked with group III strains was found to be 5 spores/g and ~250 spores/g for type E. This LOD is consistent with previously published results regarding other matrices or even lower. Woudstra et al. (2012) found a LOD of less than 50 spores/g of spiked cecum samples using a method combining an enrichment step followed by DNA extraction and PCR. Lindstrom et al. (2001) found a LOD of 1000 type E spores/g of feces using a three-day enrichment step followed by conventional PCR. Although the method was not developed for type E strains, mostly due to the lack of available naturally contaminated samples, its LOD was evaluated for type E spores. Type E avian botulism occurs yearly in waterfowl around the Great Lakes area in North America and several outbreaks have already been reported in France [11]. Livers have been shown to contain *C*. *botulinum* type E spores during avian outbreaks [6], demonstrating the relevance of this matrix for botulism diagnosis. Therefore, the screening of BoNT type E is included in the avian botulism diagnosis surveillance scheme.

Evaluation of the diagnostic specificity and sensitivity of the method was determined by comparing results obtained with the method developed in this study and PCR methods developed by Kouguchi et al. [17] and Hill et al. [16]. Samples detected by the PCR developed by Hill et al. [16] and not by our method were detected with a late Ct (above 37). This was also observed in the study conducted by Woudstra et al. [4]. When considering the results at the outbreak level and not at the individual sample level, only two suspected outbreaks tested positive with Hill method and negative with our method. For one suspected outbreak, only two livers were analyzed and not four as recommended [3]. Regarding the second suspicion, the mouse bioassay was additionally performed with sera collected on birds was negative for BoNT C, D and E. Ionophore intoxication was highly suspected to explain the observed mortality. Therefore, the method developed here is specific (100%) and sensitive (95.35%).

As recently reported, mosaic BoNT type C/D (76%) is the most common BoNT type associated with avian botulism in France [3, 4, 35]. However other BoNT types were identified, such as types D and D/C in turkey botulism outbreaks. Interestingly, almost half of the confirmed outbreaks in turkeys (46.7%) were type D or D/C. Although broilers have been described to be resistant to type D BoNT [36], turkeys appear to be sensitive to this BoNT type. Turkeys are known to be more sensitive to BoNT type C than broilers [37]; this seems to be also the case for the other BoNT types. BoNT D and D/C are highly frequent in bovine outbreaks [4, 38] and type D/C has been shown to be adapted to bovine neuroreceptors [39]. To date, no study has been conducted to evaluate the toxicity of BoNT type D and D/C on turkeys and the adaptation of BoNT type D/C to turkey neuroreceptors should be investigated.

Mallard duck was the most common species diagnosed among wild birds during this study. Ducks are known to be sensitive to botulism and to be one of the species the most affected by avian botulism [40, 41]. We also diagnosed two outbreaks involving swans. Large outbreaks involving swans have been reported in the literature, illustrating the sensitivity of this species to botulism [42].

Two pathways have been described for avian botulism: ingestion of preformed BoNT from carcasses or maggots and toxicoinfection, associated with *in situ* production of BoNT by *C*. *botulinum* in cecum and ingestion of BoNT from cecal droppings [2]. The role of each pathway among avian botulism outbreaks is unknown but they may likely coexist [2]. The presence of *C*. *botulinum* in liver birds in outbreaks investigated here suggests that the toxicoinfection pathway was involved in the investigated outbreak. This does however not indicate that the other pathway was not also involved.

The global method developed and validated in this study is highly specific and sensitive. Moreover, the results can be produced without special training for laboratory technicians and in a shorter time than previously published PCR based-methods (24 h of enrichment, 4 h for DNA extraction and 75 min for real-time PCR) [4] or than the mouse bioassay. This method has been applied to diagnose avian botulism in France for two calendar years: it confirmed 22 outbreaks, among which the type C/D was the most common BoNT type responsible for avian botulism.
